# Toxicity from Metals, Old Menaces and New Threats

**DOI:** 10.3390/ijerph7124278

**Published:** 2010-12-20

**Authors:** Wayne Briner

**Affiliations:** Department of Psychology, University of Nebraska at Kearney, Copeland Hall, Kearney, NE 68849, USA; E-Mail: brinerw@unk.edu; Tel.: +1-308-865-8196; Fax: +1-308-865-8980

Metals make up the bulk of the periodic table and range from the very light (e.g., beryllium) to the very heavy (e.g., the actinides). Metals are important constituents of life, drive economic activity and industry, but can also be a hazard to human health. The metals can be roughly divided into three groups. The first being those metals, such as iron and zinc, that are essential to human life and have a wide therapeutic dose range. The second group of metals, such as lead, mercury, and uranium, has no known biological role and are toxic even at low doses. The third group of metals, such as selenium and manganese, has a role in maintaining human health but has a very narrow dose range that, when exceeded, produces toxic effects.

A quick look at the periodic table also shows us that there is a very rough correlation between the atomic weights of metals and their toxicity. The heavier the metal the more likely it is to be toxic, and while there are exceptions (for example, beryllium is very light and also very toxic) this rough correlation has driven our view of the relative risk of metals for many years. The approach of using atomic weight as a guide to toxicity seemed to work in the period of early industrialization when lead and mercury were being used in abundance and this concept was reinforced with the birth of nuclear power when heavy metals were again seen to be very toxic and, additionally, radioactive.

In contemporary society the correlation between metal toxicity and atomic weight is no longer useful [1]. For one, we have come to understand that many of the lighter metals, such as aluminum, can be toxic. Additionally, industrial processes have moved new metals and combinations of metals into the environment. Gallium arsenide, for example, is used extensively in the microprocessor industry yet a Pub Med search yields only, at this writing, 37 articles addressing its toxicity, which in the world of toxicology means that it has mostly been ignored. Rechargeable batteries use a variety of metals to perform their function, yet despite calls for recycling, most end up in landfills with the potential to release nickel, mercury, cadmium, lithium and other substances into the environment [2]. This special edition of The International Journal of Environmental Research and Public Health has brought together authors to address a number of the issues facing those of us interested in the toxicity of metals.

The fact that the nature of metal exposure is changing can be seen even in war. Uranium toxicity has long been an issue only for the nuclear industry but as militaries across the world accelerate the use of depleted uranium munitions exposure to this once exotic metal may become much more common. I have reviewed the toxicity of this metal and made several recommendations for continued study.

The medical use of metal containing compounds is also accelerating [3]. Cisplatin and silver sulfadiazine are two well known examples of metal containing compounds. Many new metal containing drugs are being developed. Chitambar reviews the current state of gallium compounds as potential as an antibiotic and antineoplastic agents. Lanthanum is being used to control serum phosphate levels. Tin and antimony have potential to treat malignancies. With any therapeutic compound toxicity is always an issue, either because of overdose or because of the metabolic fate of the drug in question. Toxicity may be more of a concern with metal containing compounds because the elemental core cannot be subsequently metabolized. Familiarity with the metal toxicity associated with new therapeutic metal compounds will be required of the physicians using these medications. Sundar and Chakravarty review the potential therapeutic promise, and toxicity, of antimony.

Plum and her colleagues reminded that not only are some metals toxic, but in the case of some, for example zinc, deficiency continues to be an important cause of human suffering. This adds yet another layer to the way we must examine the role of metals in human life and medicine.

In a vein similar to the pharmacologic application of metal based drugs work continues on ultra trace metals and their role and toxicity in humans. These metals (e.g., molybdenum, chromium, nickel) appear to play a role in human metabolism but have very narrow therapeutic windows. Balancing the biological need for these elements with the potential toxicities can be particularly challenging. If medicinal compounds based on these metals are developed the ability to work within that narrow therapeutic window will be demanding and require a solid understanding of the toxicity of the metals involved.

While we may pay more attention to new and more exotic metals many of mankind’s old nemeses remain. Dr. Li and colleagues remind us that mercury is still a problem. Similarly Miranda and her group explore the continuing effect of lead on developing humans. Arsenic exposure has been a significant issue in the developing world for many years, a topic well explored by others [4]. These metals may also enter the food chain as a potential route of exposure. The groups of Li and Milicevic explore the expanding study of the interplay of food and metals.

Lastly, exposure to metals produces a variety of effects depending on the metal, dose, route, and time frame. Adverse effects, be they from pollutants or medications, will need to be addressed. Treatment for metal exposure may range from simple supportive care to the use of chelators to reduce metal levels rapidly. Chelation, however, is not a one size fits all strategy. The chelator selected varies with the metal involved, the relative risk of the metal, the risk of chelation, and the likely effectiveness of the chelating agent. The intricacies of chelation are discussed by Drs. Flora and Pachuari.

In many ways those of us interested in metals find this an exciting time. Therapeutic promise is on the rise while at the same time exposure to metals as pollutants is also increasing. The need to understand the actions, risks, and environmental status of metals will only grow with time. It is the hope of myself, and the authors within, that this special issue of the International Journal of Environmental Research and Public Health will help meet the demand for that knowledge.
